# Exploration of the role of *Fomitopsis officinalis Ames* in the treatment of gastric cancer using network pharmacology, molecular docking, and in vitro experiments

**DOI:** 10.3389/fphar.2025.1656365

**Published:** 2025-09-19

**Authors:** Xue Guan, Dazhong Chen

**Affiliations:** Heilongjiang University of Chinese Medicine, Harbin, China

**Keywords:** gastric cancer, Fomes officinalis ames, network pharmacology, molecular docking, apoptosis

## Abstract

**Background:**

Gastric cancer (GC) is one of the most common malignant tumors worldwide, with approximately one million people diagnosed with gastric cancer each year. Although the incidence and mortality rates of gastric cancer have decreased in recent years, it remains the third leading cause of cancer-related deaths globally. *Fomes officinalis Ames* (FOA), also known as A LI HONG or deciduous matsutake, the dried fruiting body of medicinal polypore fungus belongs to the Polyporaceae family. It is traditionally used in Chinese medicine to relieve coughs and asthma, dispelling wind and dampness, reducing swelling, and alleviating pain. However, the role of FOA in the treatment of gastric cancer remains unknown. This study systematically elaborates on the therapeutic effect of FOA triterpenic acids on human gastric cancer MKN-45 cells, providing a theoretical basis for their development as natural-sourced anti-tumor drugs. Future research could further explore their molecular targets, tap into the application value of FOA triterpenic acids in the comprehensive treatment of GC, and offer new strategies for the integrated traditional Chinese and Western medicine treatment of GC.

**Methods:**

In this study, a network pharmacology approach was employed to identify relevant targets from drug and disease-related databases. The five active components of FOA were retrieved from the Swiss Target Prediction (http://www.swisstargetprediction.ch/) database, and 379 drug active component targets were predicted. A total of 14,092 gastric cancer targets were retrieved from the GeneCards (https://www.genecards.org/) database using the keyword “gastric cancer”. Venny 2.1 was used to analyze the intersections, and a protein-protein interaction (PPI) network was constructed. The PPI network contains 328 nodes and 4,486 edges. Core targets were visualized and analyzed using Cytoscape software. Subsequently, Gene Ontology (GO) and Kyoto Encyclopedia of Genes and Genomes (KEGG) enrichment analyses were conducted to elucidate the associated biological functions and signaling pathways. Meanwhile, molecular docking of core targets with the main chemical components of FOA was performed using AutoDock software. Finally, *in vitro* experiments were conducted using human gastric cancer MKN-45 cells to validate the findings.

**Results:**

Network pharmacology and molecular docking studies have shown that the triterpenic acid components in FOA exhibit strong molecular binding affinity with the core targets CASP3 and EGFR. They also predict that FOA may exert anti-gastric cancer effects by regulating pathways such as neuroactive ligand-receptor interaction and cancer-related pathways. Additionally, a method for extracting and purifying FOA triterpenic acids was successfully established, with its methodological validation meeting the required standards; chromatographic peaks of triterpenic acid indicator components were observed at the corresponding retention times (4.37 min, 16.71 min). *In vitro* experiments demonstrated that Fomitopsis officinalis polysaccharides and triterpenic acids could significantly inhibit the proliferation of gastric cancer cells. The IC50 after 24 h and 48 h of treatment were 41.26 μg/mL and 16.21 μg/mL, respectively. These components arrested the gastric cancer cell cycle at the G1, inhibited cell migration and invasion, and induced cell apoptosis by activating CASP3. All these findings further highlight the potential application value of FOA in gastric cancer treatment.

## 1 Introduction

Gastric cancer (GC) is one of the most common malignant tumors in Asia and globally, and it is the highest-incidence digestive tract malignancy in China, with mortality rates ranking third (Thrift and El-Serag, 2020). The onset of gastric cancer is a complex process involving multiple factors, multi-gene regulation, and participation in multiple steps. Environmental factors, dietary habits, lifestyle, and pathogens such as *Helicobacter pylori* infections all contribute to the development of gastric cancer (Foqiang et al., 2023). The primary pathogenesis mechanisms are also highly complex, making it one of the significant risk factors threatening human health. Although current treatments for gastric cancer include surgery, chemotherapy, radiotherapy, targeted drugs, and immunotherapy (Yan et al., 2021), most cases are detected at an advanced stage when the cancer has already metastasized and spread, often making surgical intervention impossible. At this point, only drug therapy can be used to maintain the patient’s condition. With the continuous deepening of research on traditional Chinese medicine, natural products extracted from medicinal herbs have been found to possess low toxicity and fewer side effects while exhibiting anti-tumor properties (Zheyu et al., 2017). Therefore, the isolation and extraction of single active compounds from traditional Chinese medicine for treating GC hold great potential for future development.

*Fomes officinalis(Vill.ex Fr.)Ames* from the Fungi kingdom, belonging to the genus Hymenochaete in the Polyporaceae family. It is the dried fruiting body of the medicinal fungus Hymenochaete rickii, and is mainly found in regions of China such as Heilongjiang, Jilin, Inner Mongolia, and Xinjiang. It is a traditional ethnic medicinal material with high pharmaceutical value. According to Xinjiang Uyghur traditional doctors, Fomes officinalis Ames has a drying and warming nature, with functions such as warming the stomach, resolving phlegm, regulating qi, alleviating asthma, dispelling wind and dampness, and reducing swelling and promoting diuresis. It is commonly used to treat chronic bronchitis and various types of cancer (Zhang et al., 2014). According to literature, triterpenoid components from FOA can increase the spleen index in tumor-bearing mice and elevate the IL-2 levels to varying degrees (Chi and Jia, 2016), suggesting that its anti-tumor effects are related to enhancing the body’s immune function. Additionally, extracts from FOA can inhibit tumor cell proliferation by affecting the progression of the tumor cell cycle (Li et al., 2013a). Triterpenic acids from FOA can also induce apoptosis by increasing reactive oxygen species (ROS) levels and modulating the expression of Bax and Bcl-2 proteins. This leads to the opening of the mitochondrial permeability transition pore (MPTP), resulting in a decrease in mitochondrial membrane potential. The reduction in mitochondrial membrane potential causes relative hypertonicity within the mitochondria, leading to matrix swelling, outer membrane rupture, and the release of cytochrome C. This activates the caspase cascade reaction, ultimately resulting in the activation of caspase-3 and induction of apoptosis (Zhang, 2013). The Triterpenic acids from FOA demonstrate significant efficacy in the prevention and treatment of various tumor diseases, particularly showing remarkable preventive effects against liver cancer, gastric cancer, and colon cancer (Shi et al., 2017). Researchers have reported that an ether-extracted extract from FOA inhibits the proliferation of human liver cancer cells SMMC-7721, gastric cancer cells SGC-7901, and laryngeal carcinoma epithelial cells HEP-2 *in vitro* (Li et al., 2013a).

Network pharmacology, as a systematic approach to identifying disease-drug-target relationships, helps broaden our understanding of drugs and their biological and pharmacological effects, as well as the mechanisms by which drugs act within disease networks (Zhu et al., 2024). In this study, we employed a network pharmacology approach using databases such as HERB and SwissTargetPrediction to screen for the effective components and potential targets of FOA. We then intersected these targets with gastric cancer-related genes to identify possible targets of FOA’s active components in gastric cancer. The intersecting targets underwent Gene Ontology (GO) functional enrichment analysis and Kyoto Encyclopedia of Genes and Genomes (KEGG) pathway enrichment analysis, followed by visualization to identify core pathways. Subsequently, molecular docking was performed to further investigate the interactions between key targets and active components, elucidating their inhibitory mechanisms in gastric cancer and revealing the potential of FOA in treating the disease. *In vitro* experiments validated the anti-cancer effects of FOA on human gastric cancer MKN-45 cells and confirmed the critical targets involved.

This study is the first to combine network pharmacology, molecular docking, and experimental validation to systematically elucidate the relevant pathways and targets of FOA in the treatment of GC. To further verify the bioinformatics research findings, we conducted *in vitro* experiments using human gastric cancer MKN-45 cells. These experiments included proliferation and migration assays, invasion assays, cell cycle analysis, apoptosis assays, and core protein Caspase-3 analysis. These findings reveal that FOA possesses a unique ability to regulate tumor cell apoptosis, providing a theoretical foundation for its potential clinical application as a novel anti-gastric cancer drug. In summary, this study is expected to provide theoretical support for the clinical application of FOA in gastric cancer treatment and contribute to exploring the potential of FOA as a novel formulation for gastric cancer.

## 2 Materials and methods

### 2.1 Screening of potential targets for FOA against GC

We used the HERB (http://herb.ac.cn/) database to retrieve all chemical components of FOA by searching for “A LI HONG. “The PubChem IDs of all components were then copied into the PubChem (https://pubchem.ncbi.nlm.nih.gov/) database, In this way, the structural information of the compounds could be obtained. We screened primary components using gastrointestinal (GI) absorption and Lipinski’s Rule of Five (miLogP ≤5, nOHNH ≤5, nOH ≤10), then further identified effective active components via the SwissADME database, incorporating additional criteria: GI absorption, and Ghose, Veber, Egan,and Muegge rules. After applying these filtering conditions, we identified a total of five effective active components from FOA. Next, we used the Swiss Target Prediction database to predict the targets of these selected active components, resulting in 379 drug-target interactions. Using “gastric cancer” as a keyword, we searched the GeneCards database and obtained a total of 14,092 gastric cancer-related targets. Drug and disease targets were used to draw a Venn diagram on the SRplot (http://www.bioinformatics.com.cn/), and 329 drug-disease common targets were obtained after taking their intersection.

### 2.2 Construction of drug-disease common targets and PPI interaction network

These overlapping genes represent potential therapeutic targets for drug-disease treatment. The intersecting targets between FOA and gastric cancer (GC) were then inputted into the STRING (https://string-db.org/) database for multi-protein interaction searches. We set the species as “*Homo sapiens*” and the interaction threshold at 0.9 to obtain a more tightly connected protein network with higher confidence. This process resulted in the construction of a protein-protein interaction (PPI) network, which included 328 nodes and 4,486 edges. Subsequently, we used Cytoscape 3.10.3 software to visualize and perform network topology analysis on this PPI network. Isolated nodes (genes) were removed from the network. In the resulting network, the larger and darker-colored nodes indicate higher degree values (i.e., more connections), suggesting that these targets play critical roles within the network.

### 2.3 GO functional enrichment and KEGG pathway enrichment analysis

The collected common targets were imported into the DAVID (https://david.ncifcrf.gov/) database to perform Gene Ontology (GO) functional enrichment analysis and KEGG pathway enrichment analysis, followed by visualization. The results were sorted by p-values for cellular components (CC), molecular functions (MF), and biological processes (BP). The data were downloaded, and the top 10 core targets were selected. These targets were then imported into the MicroSignal platform for GO analysis, generating bubble plots, and KEGG pathway analysis.

### 2.4 Molecular docking

First, five active ingredients were retrieved from the PubChem database and saved in SDF format. Chem3D 2020 software was used to optimize the molecular structure of these small molecules via molecular force field optimization, resulting in the lowest energy state optimal molecular structures. Five core targets closely related to these five active ingredients were identified: AKT1 (PDB ID: 1H10), SRC (PDB ID: 1A07), EGFR (PDB ID: 1M14), BCL2 (PDB ID: 1QX3), and CASP3 (PDB ID: 1GFW). To preliminarily validate the network prediction results, the five main active ingredients and the five core targets were subjected to molecular docking using AutoDock Vina 3.10.3. A binding affinity of less than −7.00 kcal/mol indicated satisfactory binding strength. The top six molecular docking binding energies were selected for visualization analysis using PyMOL.

### 2.5 Preparation of FOA triterpenic acid extract

Take an appropriate amount of FOA medicinal material in chunks, and reflux extract it twice with 10 times its volume of 90% ethanol for 2 h each time. Combine the extraction solutions, filter them, and concentrate under reduced pressure to 1/5 of the original volume. This gives the crude extract of FOA triterpenic acid. Load the crude extract into a certain volume of pretreated D101 macroporous adsorption resin. Apply the previously prepared sample solution onto the column at a flow rate of 2 mL/min, and elute with 90% ethanol. After collecting the eluate, evaporate the solvent to obtain the purified FOA triterpenic acid. The contents of dehydrosulphurenic acid and dehydroeburicoic acid were used as evaluation indicators. The preparation method was finally determined by HPLC analysis. The results showed that the method is feasible and the process is stable and reliable.

### 2.6 Cell culture and cell proliferation assay

Human gastric cancer MKN-45 cells were purchased from Zishan Biotechnology Co., Ltd. The cells were cultured in RPMI 1640 medium supplemented with 10% fetal bovine serum (FBS) and 1% penicillin-streptomycin at 37 °C in a humidified incubator containing 5% CO2. The effect of FOA triterpenic acid extract on cell proliferation was evaluated using the CCK-8 assay. The highly sensitive cell proliferation detection reagent (CCK-8) was purchased from Yacoen Biotechnology Co., Ltd. and used to assess cell viability in response to different concentrations of FOA triterpenic acid extract. Various concentrations of FOA triterpenic acid extract (0, 7.5, 15, 30, 60, 120 μ g/mL) were added to the respective groups to evaluate cell viability. The control group (0 μg/mL) received RPMI 1640 medium supplemented with 10% FBS. After treatment with FOA triterpenic acid extract for 24 and 48 h, 10%CCK-8 cell proliferation-toxicity detection reagent was added to each well, and the plates were incubated at 37 °C for 2 h.

The optical density (OD) was measured at 450 nm using a microplate reader. The half-maximal inhibitory concentration (IC50) of FOA triterpenic acid extract on MKN-45 cells was calculated based on these measurements.

### 2.7 Scratch assay and transwell assay to detect cell migration ability

A sterile 200 μL pipette tip was used to create straight lines across the cell monolayer. After scratching, the old culture medium was discarded, and the cells were washed with PBS buffer. The starved cells in each group were treated with different concentrations of FOA triterpenic acid extract (20, 40, 80 μg/mL) for 24 h, while the control group (0 μg/mL) received RPMI 1640 medium supplemented with 10% FBS. Microscopic images were taken immediately after adding the treatment (time recorded as 0 h). The cells were then returned to the incubator for further culture. After 24 h, additional microscopic images were taken to observe the migration of the cells.

Transwell Assay experiment was divided into a control group (0 μg/mL) and three concentration treatment groups (20, 40, 80 μg/mL). Matrigel was thawed overnight at 4 °C and kept on ice during the operation. RPMI 1640 medium was mixed with Matrigel matrix at a ratio of 1:8. Then, 50 µL of the diluted Matrigel was added to the upper chamber of the Transwell insert and allowed to solidify. MKN-45 cells were seeded at a density of 5 × 10^5^ cells per insert containing the solidified Matrigel. The lower chamber was filled with 500 µL of culture medium containing 20% FBS for both the control and treatment groups. The cells were incubated for 48 h. After 48 h, the upper chambers were removed, and the inner side of the membrane was gently wiped with a cotton swab to remove non-migrated cells. The membranes were washed twice, fixed, stained with crystal violet, washed again, and air-dried upside down. The number of MKN-45 cells that had migrated through the Matrigel to the outer side of the membrane was observed under a microscope. Three clear fields were randomly selected for imaging and cell counting.

### 2.8 Flow cytometry to detect cell cycle and apoptosis

After treating the cells with different concentrations of FOA triterpenic acid extract (20, 40, 80 μg/mL) for 24 h, the control group (0 μg/mL) was also collected for flow cytometry analysis. The cell cycle detection kit (2024R1E) and the cell apoptosis detection kit (2024F2K) were purchased from Beijing Pulilai Gene Technology Co., Ltd. For cell cycle and apoptosis analysis, MKN-45 cells were stained according to the manufacturer’s instructions. Specifically, the cells were processed using the respective kits and then analyzed using a small flow cytometer-3F-3 (Apogee, United Kingdom).

#### 2.8.1 Detailed steps

Cell Collection: After treatment with different concentrations of FOA triterpenic acid extract for 24 h, both treated and control groups were collected.

Cell Cycle Analysis: Use the cell cycle detection kit (2024R1E) to stain the cells following the manufacturer’s protocol. Analyze the stained cells using the small flow cytometer-3F-3 (Apogee, United Kingdom) to determine the distribution of cells in different phases of the cell cycle (G0/G1, S, G2/M).

Apoptosis Analysis: Use the cell apoptosis detection kit (2024F2K) to stain the cells following the manufacturer’s protocol. Analyze the stained cells using the small flow cytometer3F-3 (Apogee, United Kingdom) to assess the percentage of apoptotic cells.

By performing these analyses, we can evaluate the effects of FOA triterpenic acid extract on the cell cycle distribution and apoptosis rate of MKN-45 cells.

### 2.9 Colorimetric assay to detect intracellular Caspase-3 activity

Collect cells treated with different concentrations of Fomes officinalis Ames triterpenic acid extract (20, 40, 80 μg/mL) for 48 h. The control group (0 μg/mL) was also collected. The cells were digested with 0.25% trypsin, centrifuged, and the supernatant was discarded. The cells were washed with PBS, and the supernatant was discarded again. Next, 50 μL of lysis buffer was added to resuspend the cell pellet, which was then lysed on ice for 30 min with vortexing three times for 10 s each. The lysate was centrifuged at 4 °C at 12,000 rpm for 10 min. The supernatant was carefully collected and transferred to a new tube, kept on ice until use for Caspase-3 activity detection. For Caspase-3 activity analysis, follow the instructions provided by the Caspase-3 Activity Assay Kit (G015-1-1) from Nanjing Jiancheng Bioengineering Institute. Incubate the samples at 37 °C for 4 h. When the color change was evident, measure the absorbance at 405 nm (A405) using a microplate reader. The Caspase-3 activity unit (U) was calculated using the following formula. The experiment was repeated three times.

### 2.10 Statistical analysis

Statistical analysis was performed using GraphPad Prism software (version 9). Each gradient was set with three replicates, and the results were expressed as mean ± standard deviation (SD). For data that followed a normal distribution, one-way analysis of variance (ANOVA) was used to compare multiple groups. A p-value <0.05 was considered statistically significant.

## 3 Results

### 3.1 Prediction of FOA anti-gastric cancer potential targets and construction of PPI network

**FIGURE 1:**
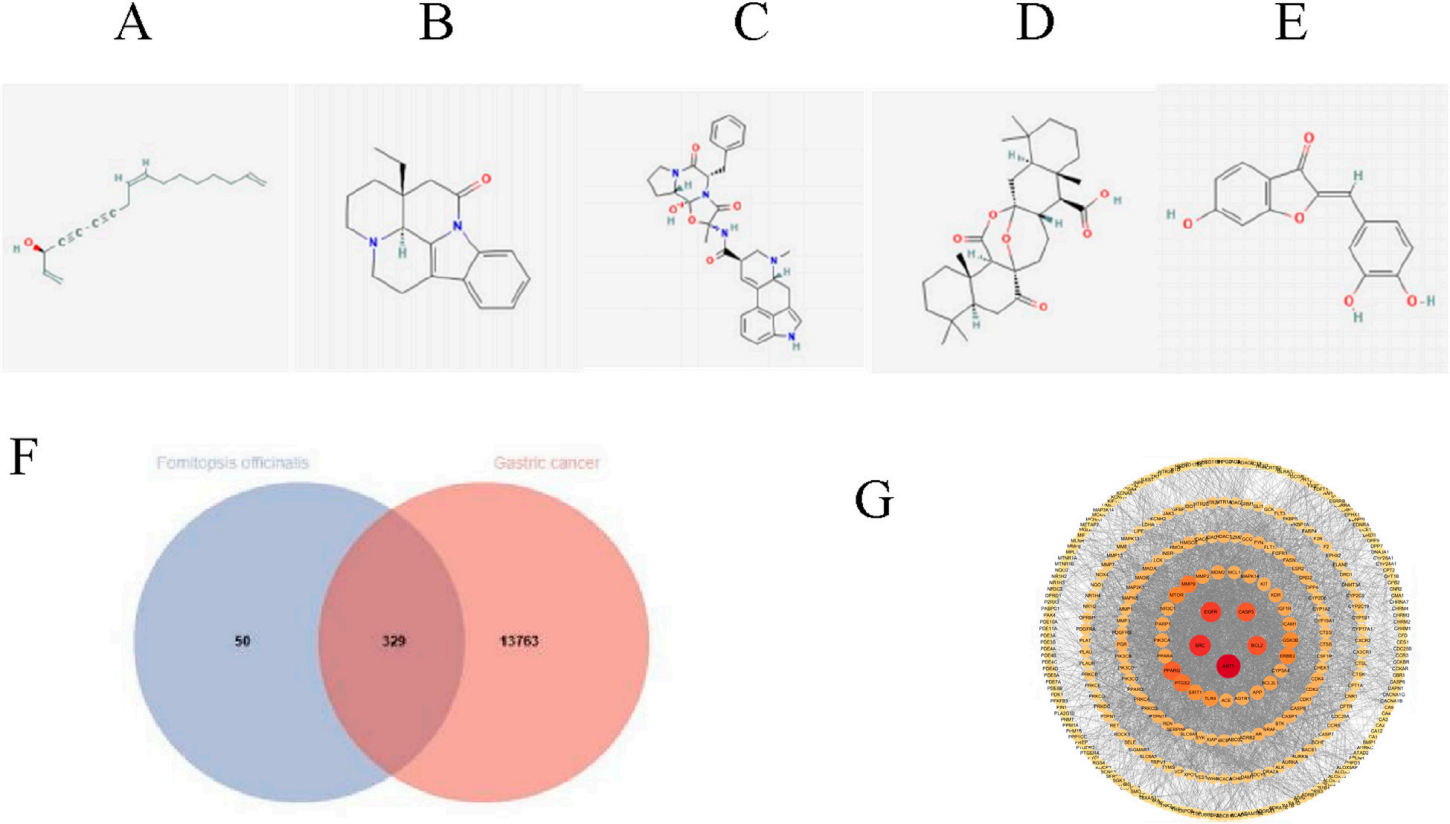
FOA-Gastric Cancer Interaction Network Analysis. **(A–E)** Chemical structures of the effective components of FOA. **(F)** Venn diagram illustrating the intersection of drug-disease targets. **(G)** PPI network displaying the core targets.

The chemical structures of the effective active components of FOA (Figures 1A–E) were obtained from the PubChem database. Gastric cancer-related genes were retrieved from the GeneCards database, and the intersection was visualized, resulting in a total of 329 intersecting genes (Figure 1F). Subsequently, the Cytoscape 3.10.3 software was used to construct the PPI (protein-protein interaction) network for these intersecting genes. In this network, nodes with deeper colors indicate higher connectivity. The top five genes with the highest degree values are AKT1, SRC, EGFR, BCL2, and CASP3 (Figure 1G), suggesting that FOA may exert its anti-tumor effects by acting on multiple targets.

### 3.2 GO and KEGG pathway enrichment analysis

**FIGURE 2:**
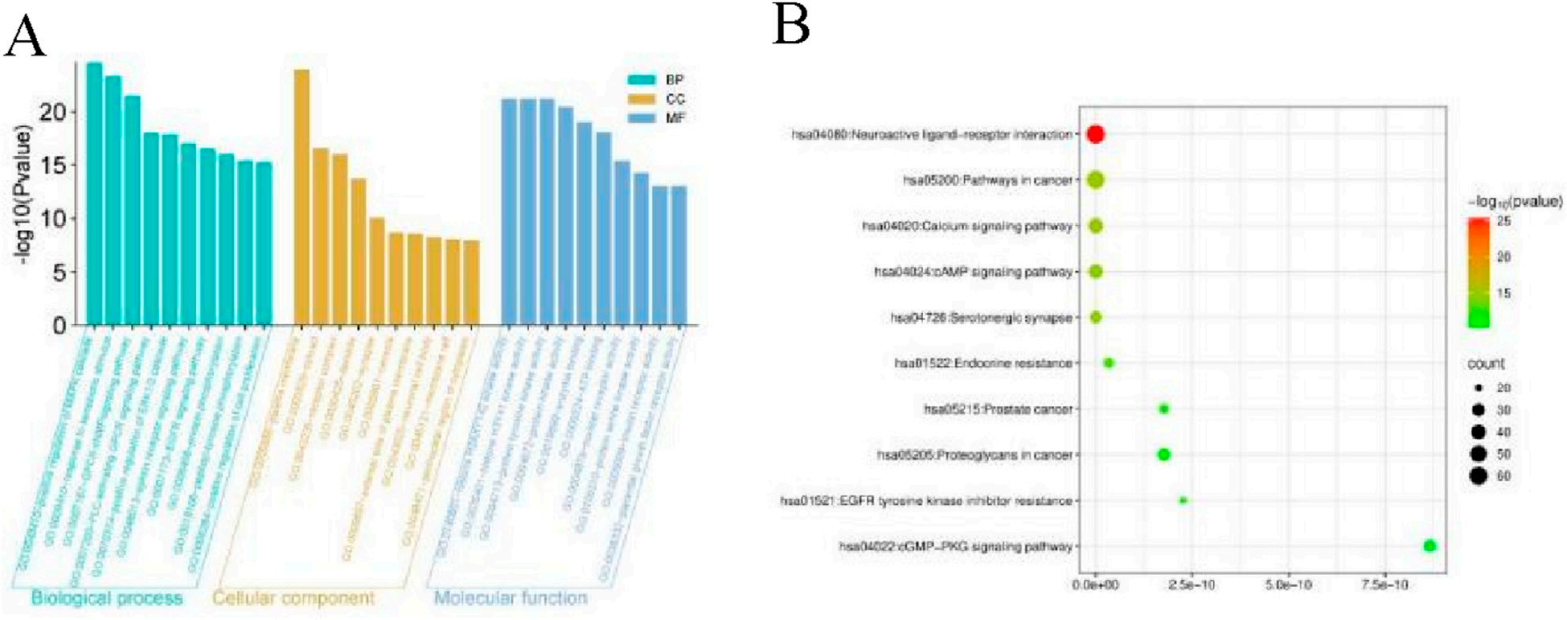
Results of GO and KEGG Pathway Enrichment Analysis. **(A)** GO enrichment results for cellular components (CC), molecular functions (MF), and biological processes (BP). **(B)** KEGG pathway enrichment analysis results.

To elucidate the role of FOA in gastric cancer treatment, we performed GO (Gene Ontology) and KEGG (Kyoto Encyclopedia of Genes and Genomes) pathway enrichment analyses on the 329 co-expressed genes using the DAVID database. GO Enrichment Analysis Results (Figure 2A). The GO enrichment analysis revealed the following biological processes associated with the core common targets of FOA-gastric cancer (ranked by relevance): Positive regulation of MAPK kinase activity, Response to external stimuli, G protein-coupled receptor-cyclic nucleotide signaling pathway, Phospholipase C-activating G protein-coupled receptor signaling pathway, Positive regulation of the extracellular signal-regulated kinase 1/2 (ERK1/2) cascade; The core drug-disease common target gene set was mainly enriched in cellular components such as: Plasma membrane, Cytoplasm, Receptor complexes, Dendrites, Synapses; The molecular functions primarily involved were histone H2AX Y142 phosphorylation activity, Histone H3 T41 phosphorylation activity, Protein tyrosine kinase activity, Protein serine kinase activity, Enzyme binding. KEGG Pathway Enrichment Analysis Results (Figure 2B). The KEGG pathway enrichment analysis selected pathways with a p-value <0.05. The representative enriched signaling pathways included neuroactive ligand-receptor pathways, cancer, the calcium signaling pathway, the cAMP signaling pathway, and serotonergic synapse.

### 3.3 Molecular docking

**FIGURE 3:**
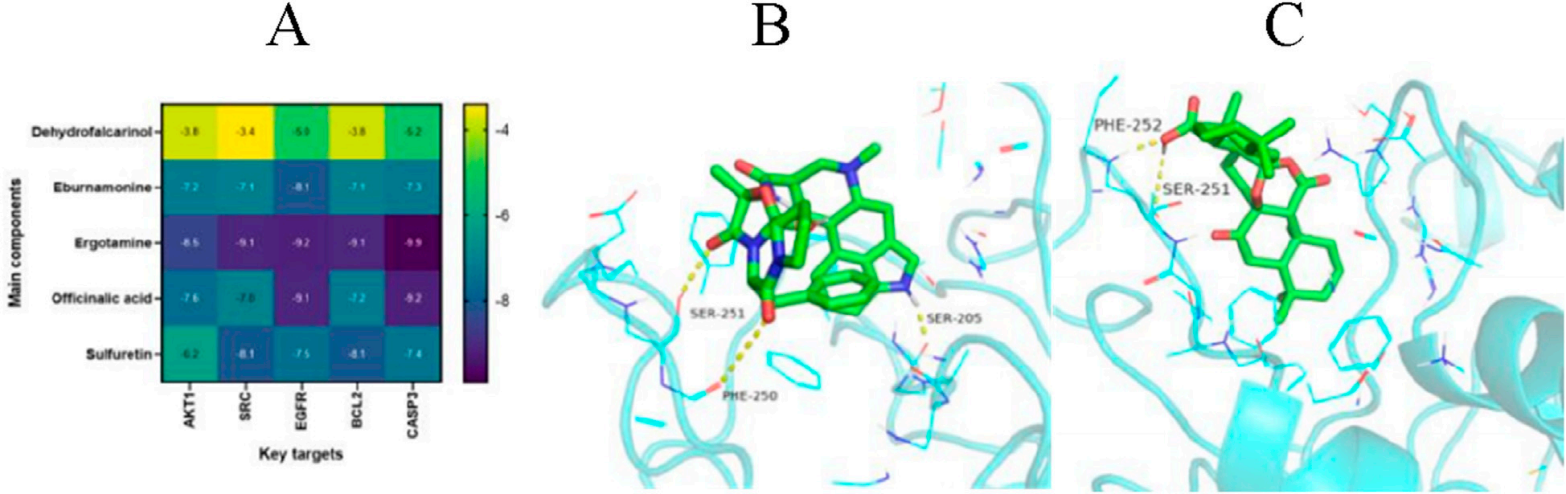
Molecular Docking Results. **(A)** Binding energies between the main active components and the core target genes. **(B)** Visualization of the Ergotamine-CASP3 molecular docking. **(C)** Visualization of the Officinalic acid-CASP3 molecular docking.

Based on the results of the PPI network analysis, five key target genes were selected for molecular docking with the five effective components of FOA. The docking results are shown in Figure 3A. The binding energies between the five target proteins and the five active components of FOA were all below −6 kcal/mol. Specifically, ergotamine formed hydrogen bonds with CASP3 (3 hydrogen bonds), EGFR (2 hydrogen bonds), BCL2 (5 hydrogen bonds), and SRC (5 hydrogen bonds). Officinalic acid formed hydrogen bonds with CASP3 (2 hydrogen bonds) and EGFR (4 hydrogen bonds). These results indicate strong binding affinities between the key active components and the core targets (Figures 3B,C).

### 3.4 HPLC analysis of index components in FOA triterpenic acid extract

**FIGURE 4:**
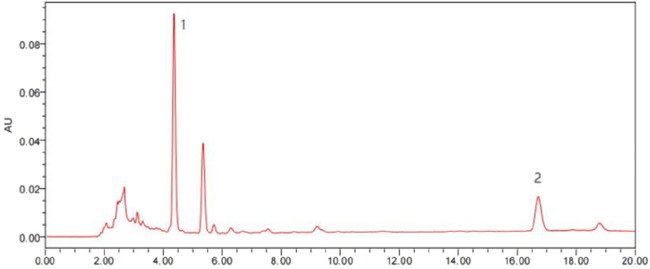
Chromatogram of FOA Triterpenic Acid Extract (1. Dehydrosulphurenic acid: Retention time = 4.37 min; 2. Dehydroeburicoic acid: Retention time = 16.71 min).

The HPLC analysis was performed using an Analytical column (4.6 mm × 250 mm, 5 μm). The mobile phase consisted of acetonitrile (A) and 0.4% phosphoric acid (B) with a gradient elution program: 80%–90% A from 0 to 8 min, and 90%–95% A from 8 to 20 min. The injection volume was 10 μL, and the detection wavelength was set at 242 nm. The flow rate was 1.0 mL/min, and the column temperature was maintained at 30 °C. The analysis results are shown in Figure 4. The retention times of the two index components were 4.37 min and 16.71 min, respectively, with well-defined peak shapes, indicating that the method is free from interference in the determination of dehydrosulphurenic acid and dehydroeburicoic acid in the FOA triterpenic acid extract. Peak 1: Dehydrosulphurenic acid (65.45 mg/g); Peak 2: Dehydroeburicoic acid (27.05 mg/g).

### 3.5 Inhibition of gastric cancer cell proliferation and migration by FOA

**FIGURE 5:**
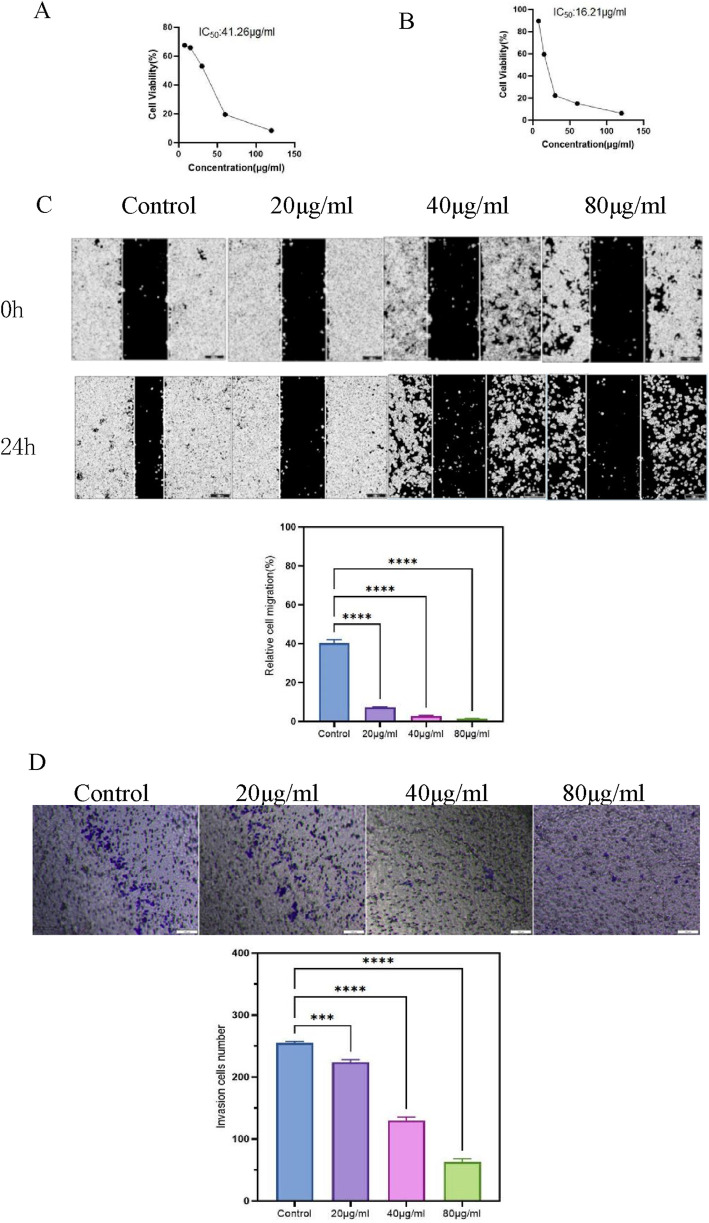
Effects of FOA Triterpenic Acid Extract on the Proliferation, Migration, and Invasion Abilities of MKN-45 Cells. **(A)** Changes in proliferation of MKN-45 cells after treatment with FOA triterpenic acid extract for 24 h. **(B)** Changes in proliferation of MKN-45 cells after treatment with FOA triterpenic acid extract for 48 h. **(C)** Effect of FOA triterpenic acid extract on the migration ability of MKN-45 cells. **(D)** Effect of FOA triterpenic acid extract on the invasion ability of MKN-45 cells.

The effect of FOA triterpenic acid extract on the proliferation of MKN-45 gastric cancer cells was evaluated using the CCK-8 assay. After treating the cells with different concentrations of FOA triterpenic acid extract (0, 7.5, 15, 30, 60, and 120 μg/mL) for 24 and 48 h, cell proliferation ability decreased with increasing drug concentration and time, indicating that FOA triterpenic acid extract inhibited theproliferation of MKN-45 cells. Additionally, the IC50 values of FOA triterpenic acid extract on MKN-45 cells at 24 and 48 h were calculated using GraphPad Prism as 41.26 μg/mL and 16.21 μg/mL, respectively (Figures 5A,B). Therefore, concentrations of 20, 40, and 80 μg/mL were selected for subsequent experiments. The wound healing assay showed that cell migration significantly decreased with increasing concentrations of FOA triterpenic acid extract (Figure 5C). Similar results were observed in the Transwell invasion assay (Figure 5D).

### 3.6 FOA induces G1 phase arrest and promotes apoptosis in gastric cancer cells

**FIGURE 6:**
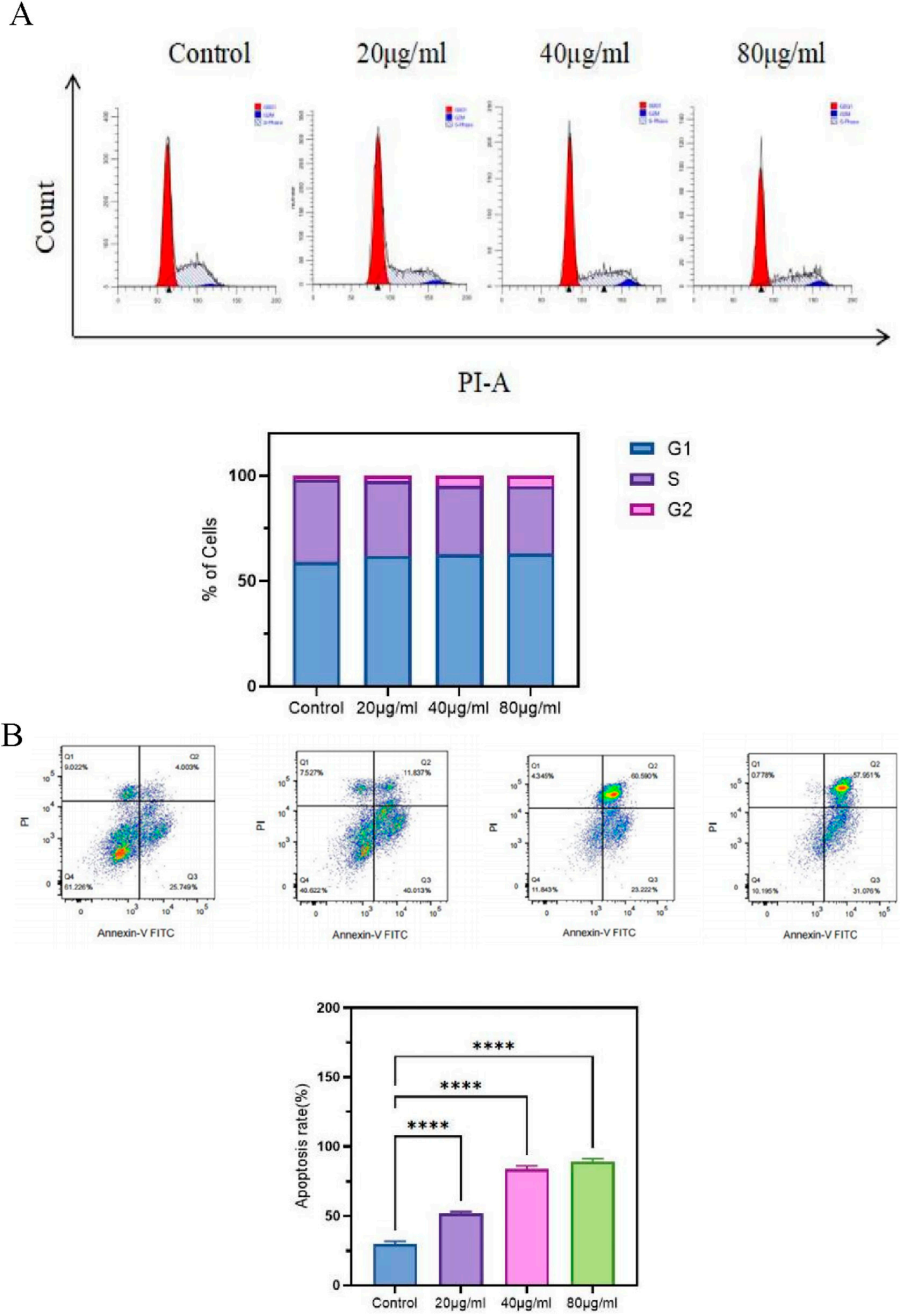
Effects of FOA Triterpenic Acid Extract on the Cell Cycle and Apoptosis of MKN-45 Cells. **(A)** FOA triterpenic acid extract induces G1 phase arrest in MKN-45 cells. **(B)** FOA triterpenic acid extract induces apoptosis in MKN-45 cells.

Additionally, gastric cancer cells were treated with FOA triterpenic acid extract at concentrations of 20, 40, and 80 μg/mL to investigate changes in the cell cycle and apoptosis. As expected, SSA (presumably a typo for FOA) induced cell cycle arrest at the G1 phase (Figure 6A) and promoted apoptosis (Figure 6B). The apoptosis rate increased with increasing concentrations of the extract. These results collectively indicate that FOA triterpenic acid extract can inhibit the proliferation of MKN-45 cells and promote apoptosis.

### 3.7 Effects of FOA on key core proteins

Caspase-3 is a crucial initiator in the death receptor-mediated apoptosis pathway (Chen et al., 2016). Caspase-3 can activate itself through oligomerization and cleavage, subsequently activating downstream cysteine proteases to produce apoptotic effects. Caspase-3 plays a significant role in inducing apoptosis and inhibiting cell proliferation. It is widely expressed in normal human tissues as well as in various tumor tissues such as lung cancer, gastric cancer, ampullary cancer, and breast cancer (Jiang et al., 2015). Experimental results showed that after treating MKN-45 cells with FOA triterpenic acid extract at concentrations of 20, 40, and 80 μg/mL, the expression levels of Caspase-3 in MKN-45 cells increased gradually with increasing concentrations. Compared to the control group, these differences were statistically significant (P < 0.001), as shown in Table 1. These findings suggest that FOA triterpenic acid extract induces apoptosis in gastric cancer cells by enhancing the expression of Caspase-3.

**TABLE 1:** Effects of FOA Triterpenic Acid Extract on Caspase-3 Levels in MKN-45 Cells (Mean ± Standard Deviation, n = 3).

Group	Dose(μg/mL)	Caspase ∼3 leves
Conrrol	0	1.82 ± 0.18
FOA triterpenic acid extract	20	3.33 ± 0.21***
FOA triterpenic acid extract	40	4.06 ± 0.09***
FOA triterpenic acid extract	80	4.67 ± 1.12***

Vs Control: ***P < 0.001.

## 4 Discussion

Gastric cancer is a highly prevalent malignant tumor of the digestive system, widespread around the world. Therefore, finding effective drugs for the prevention and treatment of gastric cancer is crucial. Triterpenoids are a class of terpenoid compounds with a basic nucleus composed of 30 carbon atoms, which can be considered as polymers of six isoprene units. They are important chemical components of natural products. One type of pentacyclic triterpenoid, betulinic acid, has demonstrated antitumor effects both *in vitro* and *in vivo*, involving more than ten types of malignant tumors, including lung cancer, colorectal cancer, and gastric cancer (Maniyamma et al., 2022). Betulinic acid is found in high concentrations in various traditional Chinese medicines such as birch bark, Ziziphi Spinosae Semen, Eucommia ulmoides, Ziziphus jujuba seeds, Abrus cantoniensis, Marsdenia tenacissima, and Prunella vulgaris (Wenkai et al., 2021). Previous studies have already confirmed that the triterpenic acid components of FOA (Friedelin-type triterpenoids) exhibit significant preventive and therapeutic effects on various types of tumor diseases, particularly in the prevention of liver cancer and colorectal cancer (Shi et al., 2017). Some researchers have reported that petroleum ether extracts from the fungus Fomes officinalis can inhibit the proliferation of human hepatocellular carcinoma cells SMMC-7721, human gastric cancer cells SGC-7901, and human laryngeal carcinoma epithelial cells HEP-2 *in vitro* (Li et al., 2013b). (Shi et al., 2017) found that the main triterpenic acid component of FOA, 3-keto-dehydrosulochrin, significantly inhibits the proliferation of human breast cancer MCF-7 cells *in vitro*. The triterpenic acids of FOA have shown inhibitory effects on the proliferation of various types of tumor cells. Additionally, *in vivo* anti-tumor experiments conducted on human lung adenocarcinoma A549 cell lines have yielded promising results. However, research on human gastric cancer cells has so far been limited to the level of cell proliferation, with no further exploration into the specific mechanisms or *in vivo* studies.

In this study, a network pharmacology approach was used to screen out five effective components of FOA (Friedelin-type triterpenoids). Using molecular docking techniques, it was found that these five effective components and their key molecular targets (AKT1, CASP3, EGFR, BCL2, SRC) all exhibit strong binding affinities. However, compounds such as Dehydrofalcarinol, Eburnamonine, Ergotamine, and Sulfuretin have not been specifically reported in the literature. In contrast, Officinalic acid, which is one of the triterpenic acid components of FOA, has been well-documented. Officinalic acid, along with other triterpenic acids from FOA, can induce apoptosis by increasing ROS (reactive oxygen species) levels and altering the expression of Bax and Bcl-2 proteins. Specifically, it increases the expression of Bax protein and decreases the expression of Bcl-2 protein, leading to the opening of the mitochondrial permeability transition pore (MPTP). This results in a decrease in mitochondrial membrane potential, causing the mitochondria to become relatively hypertonic inside, leading to swelling of the matrix, rupture of the outer membrane, and release of cytochrome C. The released cytochrome activates the caspase cascade reaction, ultimately resulting in the activation of caspase-3 and induction of apoptosis (Zhang, 2013). Therefore, we chose to measure the content of caspase-3 to validate the results of the molecular docking.

To explore the potential mechanisms of FOA (friedelin-type triterpenoids) in the treatment of prostate cancer, we conducted GO (Gene Ontology) and KEGG (Kyoto Encyclopedia of Genes and Genomes) enrichment analyses to investigate the biological functions and signaling pathways associated with FOA targets. The GO enrichment analysis results indicated that FOA positively regulates several key processes, including MAPK (mitogen-activated protein kinase) kinase activity, responses to exogenous stimuli, G-protein coupled receptor-cyclic nucleotide signaling pathways, phospholipase C activation in G-protein coupled receptor signaling pathways, and positive regulation of the extracellular signal-regulated kinases 1/2 (ERK1/2) cascade pathway. The KEGG enrichment analysis results showed that FOA primarily modulates several signaling pathways, including neuroactive ligand-receptor interactions, cancer-related pathways, calcium signaling pathways, cAMP (cyclic adenosine monophosphate) signaling pathways, and serotonergic synapses. These pathways are involved in the therapeutic effects of FOA in gastric cancer. In summary, the core common targets of FOAin gastric cancer are closely related to cancer cells at multiple levels, including biological processes, cellular components, molecular functions, and KEGG signaling pathways. At the level of biological processes, FOA participates in the regulation of key processes such as cell proliferation and signal transduction. Within cellular components, FOA is enriched at critical sites involved in material exchange and signal transduction. In terms of molecular functions, FOA involves various kinase activities and enzyme binding, which are crucial for cellular functions. In KEGG pathways, FOA covers several important signaling pathways, including neuroregulation, cancer development, and key signaling cascades. These results reveal the potential molecular mechanisms and target networks through which FOA acts on gastric cancer cells. FOA regulates multiple cellular processes, including proliferation, migration, invasion, cell cycle progression, and apoptosis, at various levels. This provides new molecular mechanisms and potential intervention targets for prostate cancer treatment.

To validate the effects of FOA triterpenic acid extract on inhibiting proliferation and promoting apoptosis in human gastric cancer MKN-45 cells, we conducted cell experiments. The results showed that FOA effectively inhibits the proliferation of MKN-45 cells. Using the CCK-8 assay to detect the inhibitory effect of FOA triterpenic acid on MKN-45 cell proliferation, we found that the half-maximal inhibitory concentration (IC50) was 41.26 μg/mL, indicating the potential of FOA as a therapeutic drug for human gastric cancer. Additionally, wound healing assays and Transwell assays demonstrated that FOA triterpenic acid inhibited the migration and invasion abilities of MKN-45 cells in a dose-dependent manner. Furthermore, FOA triterpenic acid induced G1 phase cell cycle arrest and significantly promoted apoptosis. Caspase-3 activity assays revealed that the levels of caspase-3 increased with increasing drug concentrations. These findings collectively demonstrate that the therapeutic effects of FOA triterpenic acid extract on gastric cancer are achieved through the inhibition of gastric cancer cell proliferation, migration, and invasion, induction of apoptosis, G1 phase cell cycle arrest, and elevation of Caspase-3 levels.

Our comprehensive approach combining network pharmacology, molecular docking, and *in vitro* experiments has shown that FOA triterpenic acid extract possesses anti-gastric cancer properties. These effects are mediated through the regulation of membrane receptor-mediated apoptotic signaling via the “neuroactive ligand-receptor interactions” pathway and by targeting Caspase-3 and upstream receptors/kinases to activate the apoptotic pathway. Given that Caspase-3 is a key executor of apoptosis and its encoding gene is involved in pathways regulating membrane receptor-mediated apoptosis, it is reasonable to hypothesize that FOA triterpenic acids, including officinalic acid, promote gastric cancer cell apoptosis by targeting Caspase-3.

This study provides a reference for the therapeutic mechanisms of FOA in gastric cancer, with a particular focus on the regulation of apoptosis. The research results obtained through network pharmacology, molecular docking, and experimental methods are reliable, but there are still some limitations. Further validation through *in vivo* experiments and protein-level analysis is necessary. Additionally, the potential of FOA to be developed into a novel anti-tumor drug requires further evaluation regarding its druggability. Future studies can address these limitations to further elucidate the role of FOA in gastric cancer treatment and its clinical applicability. In summary, FOA exhibits significant anti-proliferative and pro-apoptotic effects in gastric cancer by targeting key pathways and proteins. This lays a theoretical foundation for the development of FOA into a novel anti-gastric cancer drug and positions it as a promising candidate for treating gastric cancer.

## Data Availability

The raw data supporting the conclusions of this article will be made available by the authors, without undue reservation.
